# Cowpea Physiological Responses to Terminal Drought—Comparison between Four Landraces and a Commercial Variety

**DOI:** 10.3390/plants11050593

**Published:** 2022-02-22

**Authors:** Cátia Nunes, Rita Moreira, Isabel Pais, José Semedo, Fernanda Simões, Maria Manuela Veloso, Paula Scotti-Campos

**Affiliations:** 1 Unidade de Biotecnologia e Recursos Genéticos, Instituto Nacional de Investigação Agrária e Veterinária, Instituto Público, Av. República, 2784-505 Oeiras, Portugal; rita.moreira@iniav.pt (R.M.); isabel.pais@iniav.pt (I.P.); jose.semedo@iniav.pt (J.S.); fernanda.simoes@iniav.pt (F.S.); mveloso.inrb@gmail.com (M.M.V.); paula.scotti@iniav.pt (P.S.-C.); 2 Unidade de Geobiociências, Geoengenharias e Geotecnologias (GeoBioTec), Faculdade de Ciências e Tecnologia (FCT), Universidade NOVA de Lisboa (UNL), Monte de Caparica, 2829-516 Almada, Portugal

**Keywords:** cowpea, water deficit, commercial variety, landrace, productivity

## Abstract

Cowpea (*Vigna unguiculata*) is a robust legume; nevertheless, yield is always affected by drought, especially when it occurs during reproductive growth and seed filling. Considered a key crop in the effort to attain food security, and a suitable crop for a scenario of climate change, modern disregard for cowpea landraces is particularly detrimental as it causes genetic variability loss, compromising breeding efforts. To contribute to the evaluation of the cowpea germplasm, four Portuguese landraces (L1, L2, L3, L4) were compared with a commercial variety (CV) to evaluate their physiological responses to terminal drought and their inter-variation on productivity, under semi-controlled conditions. Despite no differences in relative water content (RWC) between the CV and the landraces under water deficit (WD), differences in leaf water potential (Ψ) defined the CV as having an isohydric control of stomata in contrast with anisohydric control for landraces. There was an identical decrease in the photosynthetic rate for all plants under stress, caused by both stomatal and non-stomatal limitations, namely, damages at the level of photosystem II as indicated by fluorescence measurements. Instantaneous water use efficiency (iWUE) was improved with stress in L1 and L3. Maintenance of higher relative chlorophyll content for longer periods in the CV revealed a stay-green phenotype. The slim differences observed in terms of stomatal control, iWUE and progression of senescence between the CV and the landraces under WD led to quite important differences in terms of productivity, as inferred from improved yield (number of pods and number of grains per plant). This is a clear result of pragmatic on-farm selection. On one hand it shows that small differences in stomatal responses or water saving strategies under WD may lead to desirable outcomes and should therefore be considered during breeding. On the other hand, it suggests that other traits could be explored in view of drought adaptation. These results highlight the need to preserve and characterize as many genetic pools as possible within a species.

## 1. Introduction

The higher demand for goods by our exponentially growing population causes a circular problem: increased gas emissions, water shortages and land overuse all lead to climate change, which in turn causes our most needed goods and foods to be increasingly difficult to secure. In this context, the Food and Agriculture Organization of the United Nations (FAO) recognizes pulses as key crops in the global effort to attain food security. In particular, cowpea (*Vigna unguiculata*) fits in the three dimensions of sustainable development: social, economic and environmental. It is a heat and drought resistant legume, rich not only in essential plant-type nutrients such as carbohydrates, fiber, minerals and vitamins, but also a healthy low-cost alternative/supplement to animal protein. Its ability to fix atmospheric nitrogen into the soil minimizes the need of fertilization and contributes to implementation and acceptance of rotation and intercropping techniques [1]. Moreover, cowpea supports poor communities, guaranteeing the livelihood of millions of families of the tropical and sub-tropical regions, mainly in Africa [2,3], alleviating hunger, feeding cattle and giving income to families.

Cowpea is a robust crop, surviving hot climates and dry semi-arid soils with little to no inputs [4], emerging as a suitable crop for a scenario of climate change and water shortage, which is already in progress in the Mediterranean area. However, as resilient as a crop may be, abiotic stresses always come with a production cost. Water deficit severity, duration, developmental stage at which it occurs and the genetic potential of the crop determines the detrimental effect of the stress episode [5]. Nevertheless, it always disrupts a plethora of physiological, biochemical, and genetic processes. To complicate matters, those processes are differently affected among species and even within varieties/cultivars/landraces of one species. For example, after a physiological analysis of twelve accessions of *Lotus tenuis*, the authors were able to pinpoint the two most contrasting populations in terms of water-stress tolerance [6]. The same intraspecific variability was also observed for cultivars of different Medicago species [7]. Upon drought, photosynthesis is one of the first physiological processes to be affected [8]. The induced stomatal closure decreases water loss but causes a decline in leaf CO_2_ uptake, leading to slower photosynthetic rates, altered responses to light, and eventually to metabolic impairment [9]. In three drought resistant grain legumes, moth bean, tepary bean and guar, stomatal conductance was the main limitation to photosynthesis under water deficit, and there was no evidence of non-stomatal limitations [10]. For *Medicago truncatula*, however, the observed drought responses indicated biochemical limitations to photosynthesis [11]. For cowpea, photosynthesis was shown to be affected by both stomatal and non-stomatal mechanisms, leading to yield losses that were not expressed as seed number per pod [12]. One way to classify the responsiveness of stomata to water availability is through the iso/anisohydric classification which determines the direct sensitivity of leaf Ψ to soil Ψ. Isohydric plants maintain steady leaf Ψ through tight stomata control in response to water availability, with consequent reduction in gas exchange under water deficit conditions. On the contrary, anisohydric plants have a looser control of transpiration by maintaining stomatal opening for longer under WD, allowing for higher rates of photosynthesis than isohydric plants under the same conditions, but risking cavitation and death [13]. Other mechanisms to tolerate drought involve processes at the cellular level, particularly antioxidant activity and osmotic adjustment. Grass pea was shown to be more drought tolerant than pea due to the accumulation of osmoprotectants and improved oxidation resistance [14]. In cowpea, the accumulation of proline is well documented [15], with the particularity of a non-uniform accumulation with preferential proline accumulation in the upper tier of the plant [16]. Other species, such as lupin, rely on more extreme mechanisms such as programed cell death and metabolic rearrangement. During water stress, lupin plants lose most leaves and the stem functions as a storage organ for sugars and amino acids, which are mobilized once the stress recedes to produce new leaves [17]. These complex and quantitative drought response traits, together with their low heritability due to high environment × genotype interactions, many times hamper selection and breeding success [18].

Farming of cowpea by traditional farmers over centuries in specific edapho-climatic areas gradually originated local cowpea populations that became landraces particularly well adapted to those local conditions [19]. Landraces are genetically rich populations that enclose valuable genetic diversity ready to be introduced in breeding programs [20]. In Portugal there are numerous landraces. The selected four, to our knowledge, were never characterized in terms of response to water deficit, remaining a potential source of variation for this important trait. As an effort to preserve and alert about the importance of on-farm conserved landraces, we used four landraces of traditional significance from the central region of Portugal which, despite the efforts of locals to maintain commercially available, are in danger of disappearing from the field. L1 (BPGV13100) was kindly provided by the Banco Português de Germoplasma Vegetal and had been originally collected from Guarda do Douro; L2 was obtained directly from a farmer at Sátão; L3 was bought at a farmer’s market and is traditionally cultivated in the area of Lardosa; L4 was also obtained directly from farmers at Vila Maior. Our aim is to determine how these Portuguese on-farm-selected landraces behave in response to water deficit (a common and aggravating condition) in comparison to a commercial variety “Fradel” developed at INIAV-Elvas under the Mediterranean climate to have improved characteristics for the farmers such as indehiscent pod and better productivity. To evaluate their inter-variation strategies towards WD they were evaluated through gas exchange and chlorophyll a fluorescence measurements, as well as through water relations and yield determinations under terminal drought. The different physiological responses were examined against yield parameters obtained under greenhouse conditions and discussed in terms of breeding usefulness.

## 2. Results

### 2.1. Leaf Water Status and Membrane Injury Index

**Figure 1:**
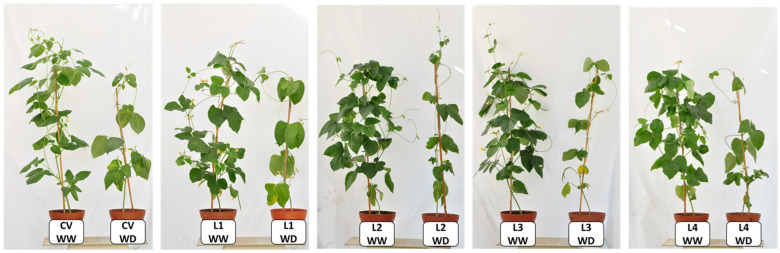
Representative morphological aspects of four cowpea landraces and a commercial variety grown in semi-controlled conditions, under well-watered (WW) and water deficit (WD) conditions, at early fruit development, between T1 (end of flowering) and T2 (end of fruit development).

Soil water content (SWC) was monitored regularly to impose two water regimes. The SWC was found to be ideal between 65%, well-watered (WW) treatment, and visible symptoms of drought were observed when the SWC was adjusted to 35% (WD treatment). The induction of water shortage began at the onset of the flowering stage (T0, BBCH 5) on 5-week-old plants. The first visible indicator of water deficit in plants was leaf wilting, reflecting the change in their internal water status, followed by mature leaf yellowing and shedding (Figure 1). Visually, the only difference observed among varieties was the maintenance of green color for longer under WD after fruit development (T2) in the CV and earlier senescence of L4 under WW conditions. Plants under WW conditions maintained about 90% RWC whereas under the imposed terminal WD, RWC dropped to about 80% (Table 1). 

Genotypes showed limited damage at the membrane level under WD, as inferred from low injury index (I%) values. Although it is a parameter that already shows some variation among varieties under WW conditions, all landraces presented small but significant increases under WD. The CV did not show this behavior due to an already elevated I% under WW conditions (Table 1).

**Figure 2:**
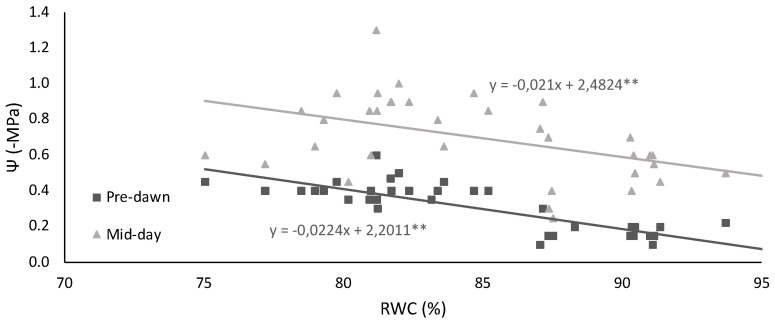
Effect of decreasing relative water content (RWC) on the water potential (Ψ) at mid-day (Ψ_MD_) and pre-dawn (Ψ_PD_) on a commercial variety (CV) and four landraces (L1, L2, L3, L4) of cowpea under well-watered and water deficit conditions. Values represent individual measurements. The presented linear regressions are the best fit for the data groups (one CV and four L of cowpea) (*p* < 0.05). ** Regression coefficient significant with *p* < 0.05.

The imposed water regime is also clearly indicated by Ψ_PD_ (Table 1) when the water flux through the system soil–plant atmosphere is low, and an equilibrium is reached. All WD plants showed significantly lower Ψ_PD_ than WW plants which had much higher values, above −0.20 MPa. At mid-day, not only in the WD but also in the WW plants, the Ψ_MD_ decreased drastically, reflecting the demand for water flux caused by the atmospheric conditions. Both parameters showed a significant linear relation with RWC (*p* < 0.05), both with identical slope (*p* < 0.001) (Figure 2).

The iso/anisohydric classification determines the direct sensitivity of leaf Ψ to soil Ψ. Estimates obtained using the model described by [21], where the degree of isohydricity is defined as the slope (σ) of the relationship between Ψ_MD_ in response to soil water content (or Ψ_PD_ as a proxy of soil water content since transpiration is strongly reduced during the night and an equilibrium is reached between soil and plant), revealed that the CV has a different behavior from the landraces. With a correlation slope below 1, the CV presents a partial isohydric strategy, whereas the four landraces have a more anisohydric behavior with a slope higher than 1 (Table 1).

### 2.2. Photosynthetic Function and iWUE

**Figure 3:**
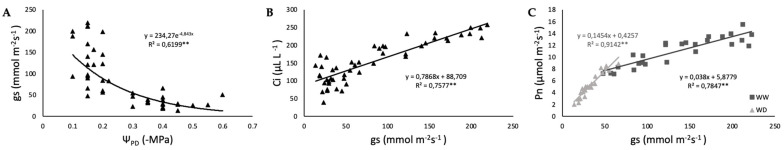
Effect of decreasing leaf pre-dawn water potential (Ψ_PD_) on leaf stomatal conductance (gs) (**A**); effect of decreasing gs on internal CO_2_ concentration (Ci) (**B**) and effect of decreasing gs on photosynthesis (Pn) (**C**) of a commercial variety (CV) and four landraces (L1, L2, L3, L4) of cowpea under well-watered (WW) and water deficit (WD) conditions. Points represent individual measurements. ** Regression coefficient significant with *p* < 0.05.

**Figure 4:**
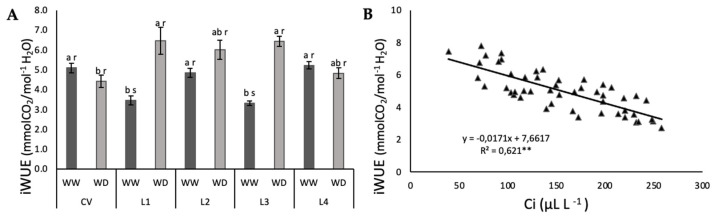
Water deficit effect on instantaneous water use efficiency, iWUE (**A**) and effect of decreasing internal CO_2_ concentration (Ci) on iWUE (**B**) of a commercial variety (CV) and four landraces (L1, L2, L3, L4) of cowpea under well-watered (WW) and water deficit (WD) conditions. Points represent individual measurements and bars represent mean ± SE (*n* = 5). Different letters mean significant differences between varieties (a,b) and between treatments for each variety (r,s) (ANOVA, *p* < 0.05; ** regression coefficient significant with *p* < 0.05).

In response to a diminished internal water content, one of the first plant responses is stomata closure (gs decrease). This parameter decreased with the imposed WD with all varieties responding similarly to Ψ_PD_ (Figure 3A) (and Ψ_MD_, data not shown), where an exponential regression is the best fit for all the data groups with all WD plants presenting a gs below 54 mmol CO_2_ m^−2^ s^−1^. The decrease of gs with stress caused a linear decrease of Ci for all plants. The presented linear regression is the best fit for all the data groups. When analyzing photosynthesis dependence on gs, there were also no differences between varieties either on WW or WD conditions (*p* < 0.05); however, there was a significant difference in the response of Pn to gs from WW to WD (*p* < 0.001) (Figure 3C). This behavior caused iWUE to be significantly higher under stress for L1 and L3, whereas no differences were observed for CV, L2 and L4 (Figure 4A).

Despite some differences in the iWUE between varieties, when analyzing this parameter in terms of response to Ci, all varieties combine in a single linear regression (Figure 4B).

**Figure 5:**
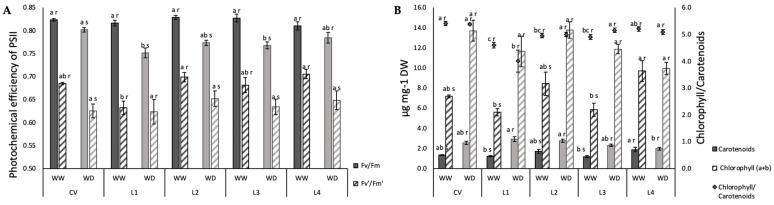
Water deficit effect on the maximal photochemical efficiency of PSII of dark-adapted leaves (Fv/Fm), and on the actual photochemical efficiency of PSII under photosynthetic steady-state conditions (Fv’/Fm’) (**A**) and on total carotenoids, total chlorophyll and chlorophyll to carotenoids ratio (**B**). Bars represent mean ± SE (*n* = 5). Different letters mean significant differences between varieties (a–c) and between treatments for each variety (r,s) (ANOVA, *p* < 0.05).

No genotypic variability was observed in WW plants with respect to the maximal photochemical efficiency of PSII of dark-adapted leaves (Fv/Fm), but WD caused a slight but significant decrease in most plants with the exception of L4 (Figure 5A). Under photosynthetic steady-state conditions, the actual PSII photochemical efficiency (Fv’/Fm’) decreased in all varieties under WD with the exception of L1, which behaved differently under WW conditions with a value lower than the remaining landraces (Figure 5A).

In terms of pigments, when determined spectrophotometrically, total carotenoid content clearly increased under WD for all plants except for L4 (Figure 5B). Nevertheless, total carotenoid to chlorophyll ratio did not change under WD because there was an accompanying increase in total chlorophyll.

**Figure 6:**
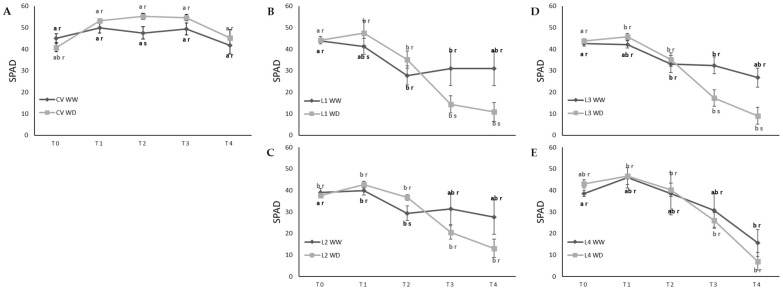
Water deficit effect on the relative chlorophyll content of leaves of a commercial variety (CV) (**A**) and four landraces of cowpea, L1 (**B**), L2 (**C**), L3 (**D**) and L4 (**E**) under well-watered (WW) and water deficit (WD) conditions, at the beginning of treatment (T0) and 5, 7, 9, 10 and 11 weeks into the treatment (T1, T2, T3 and T4, respectively). Values represent mean ± SE (*n* = 5 to 10). Different letters mean significant differences between varieties (a,b) and between treatments for each variety (r,s) (ANOVA, *p* < 0.05).

Using a non-destructive SPAD meter, relative chlorophyll content was monitored throughout the imposition of stress. Before the onset of stress, SPAD measurements showed that plants presented comparable relative chlorophyll content, with values ranging from 38.6 to 45.1 (Figure 6). Until T2, WW and WD plants followed a similar SPAD pattern. From T2 onwards, the CV maintained its relative chlorophyll content unchanged, L1, L2 and L3 suffered a shift, WW plants maintained their values whereas WD plants kept decreasing, and finally, for L4, both WW and WD plants declined similarly on their SPAD value, i.e., the CV stayed green for longer both under WD and also during development under WW conditions (Figure 6A). On the contrary, L4 senesced earlier, particularly under WW conditions (Figure 6E).

### 2.3. Leaf Sugar Content and Yield

**Figure 7:**
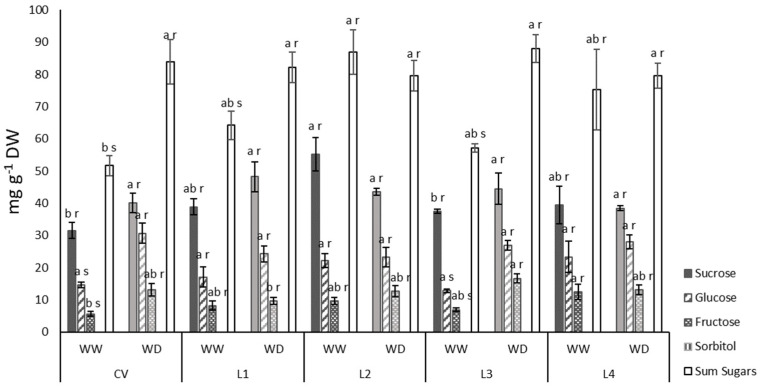
Water deficit effect on the sugar content of leaves. Bars represent mean ± SE (*n* = 5). Different letters mean significant differences between varieties (a,b) and between treatments for each variety (r,s) (ANOVA, *p* < 0.05).

The availability of sugars on the leaves, conditioned by drought-imposed constraints, should influence the amount and quality of the grain. To explore this relation, sucrose, glucose and fructose were quantified. Despite some variability of results, statistics show no relevant differences between treatments for sucrose. For glucose and fructose there was an increase under WD for the CV and L3. No differences were observed under WD between varieties. Overall, total soluble sugars increased under WD for the CV, L1 and L3, while for L2 and L4 they were kept constant. Under WW, sugar alcohols, namely sorbitol, were below detectable limits, and the imposed WD did not induce the accumulation of sorbitol (Figure 7).

**Figure 8:**
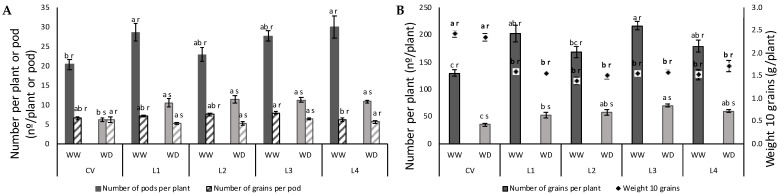

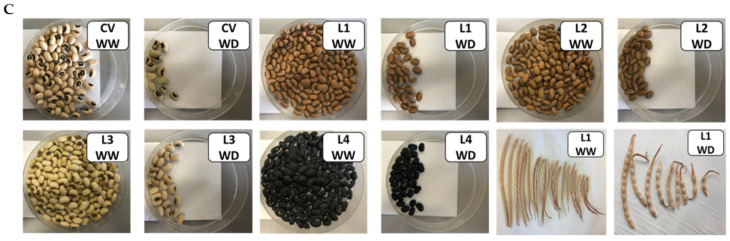
Water deficit effect on number of pods per plant and number and grains per pod (**A**), total number of grains per plant and weight of 10 grains (**B**) and grain morphology, quantity produced and pod morphology for a representative plant of each variety, under WW and WD conditions (**C**). Bars represent mean ± SE (*n* = 5). Different letters mean significant differences between varieties (a–c) and between treatments for each variety (r,s) (ANOVA, *p* < 0.05).

In relation to the obtained yield, differences are significant. All varieties suffered a large impact under WD both in number of pods per plant (Figure 8A,C) and total number of grains per plant (Figure 8B,C) with a decrease of about 60 and 70%, respectively. Number of grains per pod did not change for the CV and L4 (Figure 8A). Perhaps the most striking and unexpected difference among varieties was the lower number of pods and seed in the CV, which was still not different from L2. Nevertheless, the CV grain was significantly heavier than that of any of the landraces.

The above studied parameters were analyzed in terms of how they are affected by drought treatment and genotypes, and the interaction of these two variables (Table 2). The parameters that are more variety-associated are I%, chlorophyll a fluorescence and productivity. Considering the effect of treatment and variety together, iWUE, Fv/Fm, pigments, sugar accumulation and number of grains per plant (NGPl) are the most significant. Treatment had no effect on iWUE, Fv’/Fm’ and weight of 10 grains.

## 3. Discussion

Terminal drought refers to water shortage occurring during the reproduction phase or as late as seed filling [22] and is the most detrimental in terms of yield loss [23,24]. Varieties with improved response to this late adversity are much needed not only in dry areas that usually suffer from chronic food shortage but also in areas expected to become dryer in the coming years, such as those of the Mediterranean climate [25,26]. For now, the main method to develop crop varieties with improved resistance to abiotic stresses is, and with proven merit, conventional breeding [27]. However, this is a time-consuming method that is obstructed by low heritable genetic variability within the species [28,29] and by a deficient understanding of the physiological responses to stress (and their genetic control) that culminate in yield loss [18,30]. Preserving and studying landraces that were empirically selected to perform well in specific agro-climatic conditions may enrich the genetic pool upon which breeding programs can develop improved varieties [20]. As an effort to preserve and inform about the value of on-farm conserved landraces the present work evaluated the physiological performance under terminal drought of four Portuguese landraces in comparison with a commercial variety (developed through conventional breeding to perform well under the Mediterranean climate).

Plants under terminal drought had a small but significant decrease in leaf RWC (Table 1), denoting an ability to maintain elevated water content under stress, around 80%. The ability to maintain an elevated water content prevented strong damage at membrane level under WD, as inferred from low injury index (I%) values. RWC correlated linearly with both Ψ_PD_ and Ψ_MD_. As expected, Ψ_MD_ was significantly lower than Ψ_PD_, a reflection of the demand for water under the existing atmospheric conditions during the day (Figure 2). In grain legumes, it has been described that increased sugar alcohol (namely sorbitol and inositol) with associated decrease in soluble sugars function as strategic osmotic regulation during drought [31], a response that was not found for the studied cowpea plants, which had values of sorbitol so low that they were below the detectable limit. However, soluble sugars increased under WD for the CV, L1 and L3, suggesting that these molecules could be acting as osmoprotectants (Figure 7).

Stomatal closure is the first mechanism that plants use to maintain leaf water content within life-tolerable limits. One way to classify plants based on their approach to use this regulatory mechanism is through the isohydric–anisohydric continuum. When plants are under comparable external environments, isohydricity level has been found to be a strong predictor of the plant strategy to regulate stomata in response to water deficit [32]. Isohydric species adjust their stomata readily upon need, whereas anisohydric species have a looser control of stomata [33]. Applying the principal to our case to distinguish varieties within a species, we can say that with a correlation slope σ (of the relationship between Ψ_PD_ and Ψ_MD_) below 1, the CV presents a partial isohydric strategy whereas the four landraces have a more anisohydric behavior with a slope higher than 1 (Table 1). Despite the difficulties in the interpretation of σ when applied to different species and even more so when growing in different locations, in our case, we can assume the simpler interpretation where stomatal closure reduces transpiration to avoid steep water potential differences that could risk hydraulic failure and plant death [13]. In this sense, the CV may benefit from a tighter stomatal control, with associated disadvantages such as decreased photosynthesis due to insufficient gas exchange [34,35] and unbearable increase in leaf temperature and photo-damage under more extreme conditions [36]. On the contrary, the landraces may be more prone to hydraulic failure and irreversible wilting, but this is possibly only worrisome at more extreme water deficit, while they maintain higher photosynthetic rates at more tolerable WD levels.

The isohydric classification is not specific of each genotype, and instead shifts with growing conditions and imposed stress [37]. Nevertheless, the degree of isohydricity determines the relationship between CO_2_ assimilation and water vapor loss, i.e., the water use efficiency (iWUE). That relationship is especially critical under high atmospheric evaporative demand [38], such as our case. Most importantly, unlike isohydric level, WUE is a genetically-determined characteristic. It must therefore be explored by plant breeders [39], because an improvement in WUE would increase total biomass production as well as yield at any given level of soil water availability. In our case, only L1 and L3 show a significant increase in iWUE under WD, but when analyzed together, iWUE shows a significant relationship with Ci for all plants, in which iWUE increases with decreasing Ci. However, looking at the attained yield, L1 and L3 do not stand out alone, instead, all landraces seem to have performed better under WD than the CV, at least in terms of number of pods per plant and number of grains per plant (Figure 8). In groundnut, it was found that genotypes with higher WUE attained higher yields [40]. One could argue that the anisohydric strategy of the landraces was advantageous under the imposed conditions in comparison with the isohydric strategy of the CV.

It is well known that stomata operation is subject to feedback control, and if it is true that a certain leaf Ψ determines gs, it is also true that stomata closure, by affecting transpiration, contributes to attain a certain leaf Ψ [41]. Nevertheless, the obtained gs values correlate with Ψ_PD_ (Figure 3A) more tightly than with Ψ_MD_ (data not shown), suggesting that during the day other factors affect this relationship. On a different note, despite the predicted difference in the isohydric strategy of the CV vs. the anisohydric behavior of the landraces, there were no differences between them in terms of the response of gs to Ψ_PD_, and therefore a common regression line is presented (Figure 3A).

The first consequence of stomatal closure is Ci decrease (Figure 3B) following photosynthesis decrease due to low carbon supply [8,9,42,43,44]. An extreme reduction in the photosynthetic rate was observed for all plants (Figure 3C). The fact that the correlation between gs and Pn under WW and WD conditions is different (Figure 3C) suggests downstream effects of stress on the photosynthetic apparatus under drought. Furthermore, the decrease in Pn under WD induced by stomatal closure was steeper than the decrease observed for Ci (Figure 3B), corroborating the idea that non-stomatal limitations of photosynthesis occur during WD. For some grain legumes (such as moth bean, tepary bean and guar) not even extreme drought seems to cause non-stomatal limitations to photosynthesis [10]; for cowpea, however, both forms of limitation to photosynthesis were described [12].

Not enough intercellular CO_2_ results in the transfer of electrons through the electron transport chain to oxygen in photosystem I, releasing reactive oxygen species (ROS). If the plant is inefficient at neutralizing these ROS, photosystem II (PSII) can be damaged [8]. PSII can also be damaged by low intercellular CO_2_ if harvested light energy becomes excessive, causing photodamage. Our cowpea plants seem to be affected in this way, as both Fv/Fm and Fv’/Fm’ decreased under WD (Figure 5A), with the exception of L4 for the first parameter and L1 for the second, nevertheless showing the same tendency. This is quite different from what was observed for another water deficit-resistant legume, *Medicago truncatula*, the photosynthetic apparatus of which was kept unaltered under mild and severe water deficit [11]. In the case of cowpea, physiological aspects, mainly those related with stomatal function, and some biochemical markers seem to be more relevant during drought than antioxidant enzymatic activity, as described by [14].

Variation in chlorophyll to carotenoids ratio is a sensitive indicator of oxidative protection, allowing to infer if carotenoid molecules increase to quench excessive excitation energy that would otherwise produce reactive oxygen species. Halfway through the stress imposition, T2 to T3 (end of fruit development/beginning of ripening), the clear increase in carotenoid content indicates that there was a triggering of photoprotective mechanisms [45] under the studied WD condition, corroborating the idea that the photosynthetic apparatus could be suffering detrimental effects caused by the imposed stress (Figure 5B). The increase in carotenoids under WD was accompanied by an increase in total chlorophyl, justifying the absence of differences in chlorophyll to carotenoids ratio in all plants. L4 was the exception, with no increase in any of these parameters. As stress progresses, expected oxidative stress causes cell damage and senescence [46], as observed for the studied landraces (Figure 1 and Figure 5). These results are quite in line with those obtained by [47], who found a reduction in chlorophyll content under water stress for seven drought resistant cowpea genotypes. The fact that the CV maintained greener leaves for longer periods during development both under WW and WD conditions implies that it may be more efficient in light energy use [47,48]. This suggests some degree of resistance to water deficit at this development stage. An association between the stay-green trait and grain yield was demonstrated for cowpea under drought stress [49]. It is argued that for legumes, N-rich grain yield could be impaired by confined N at supporting the stay-green trait [50,51]. On the contrary, the stay-green trait could contribute to a higher yield by extending photosynthesis in time to feed pod filling with C-assimilates [52]. In our case, the stay-green trait of the CV did not translate into increased yield (Figure 8). Terminal water shortage in cowpea had already been shown to reduce yield parameters, with the exception of seed number per pod [53], as found for the CV and L4 (Figure 8A).

As simply put by [12], similarities of physiological responses to abiotic stress should not be assumed between related species. For example, no parallelism was observed between *V. unguiculata* and *Phaseolus vulgaris* in terms of leaf gas exchange parameters [54]. The same can be said, for example, between *V. unguiculata* and *Medicago truncatula*, in which the latter seems to have leaf photochemistry rather more resistant to water shortage [11] than what was demonstrated here for cowpea. Moreover, it has been shown that different *Vigna* species (both wild and domesticated) respond differently to non-terminal and terminal drought, and that those accessions more tolerant to one experimental condition were not the same to be found tolerant in the other. Unexpectedly, large differences in tolerance were detected in genetically close accessions [55]. Based on the present work, these assumptions seem to also be true for genotypes within a single species. Considering the effects of the drought treatment, in cowpea genotypes and their interaction for all studied parameters (Table 2), leaf water status does not distinguish varieties. All genotypes showed the same capacity to maintain tissue hydration under WD, denoting adaptation to the Mediterranean climate. Only for I% we can say that L3 has less membrane injury than the remaining genotypes, but when considering treatment, no advantage is observed. From the fluorescence parameters, the CV, L2 and L4 seem to have a slight better performance with less affected photosystems. In terms of yield, the CV stands out with bigger and heavier seeds, but L1 and L4 have higher number of pods per plant (NPPl) and L1 and L3 higher number of grains per plant (NGPl). From the interaction of genotype with treatment, the iWUE of L1 and L3 improves with WD, and these are also the landraces that show higher NGPl both under control and drought conditions. Overall, L1 and L3 presented a better performance under these study conditions. However, the differences are slight, with landraces enduring water deficit quite similarly to the CV, following a different response strategy. Nevertheless, these slim differences in the physiological response to WD led to quite important differences in terms of productivity between the CV and landraces. Considering that both the CV and landraces were selected to have the best possible yield under the Mediterranean climate, the obtained results are a clear indication of pragmatic on-farm selection. Results show that subtle differences in the underlying response to WD may lead to different outcomes. Other physiological traits could better explain the observed differences in productivity, but nevertheless it is important to be aware of these individualities during breeding decisions. These results highlight the need to preserve and characterize as many genetic pools as possible within a species.

## 4. Materials and Methods

### 4.1. Field Capacity and Water Deficit Induction

Field capacity (FC) was evaluated by gravimetric method. Pots were filled with *ca.* 3 L of peat moss soil (Arber Horticulture) and watered until saturation. After 24 h of runoff, saturation by capillarity was assured. Pots were weighed individually (approximately 1300 g), the result being considered as 100% field capacity (FC) [56].

Seeds from a commercial variety (CV) and four Portuguese *Vigna unguiculata* landraces (L1–L4) were sown in late May. The CV “Fradel” was developed at INIAV-Elvas and presents the characteristic cream grain color with black eye around the hilum; L1 was kindly given by the Banco Português de Germoplasma Vegetal (BPGV13100), the grain is clay in color with a small black eye around the hilum and had originally been collected from Guarda do Douro; L2 was obtained directly from a farmer at Sátão and the grain has the same color as L1; L3 was bought at a Farmer’s Market directly from a farmer from Lardosa, and the grain is rounder and smaller than the remaining accessions, with cream color and a light green eye around the hilum; L4 was also obtained directly from the farmer at Vila Maior and the grain is black with no visible eye around the hilum. Plants (1 plant per pot, 10 pots per landrace) were grown in a semi-controlled greenhouse and well irrigated to 80% of FC during the early vegetative growth. Water deficit (WD) was induced in 5-week-old plants by withholding irrigation in half of the plants, maintained under 35% FC. Control plants (WW) were irrigated to maintain 80% FC. Once a week, water was replaced by nutrients solution (Complesal 12-4-6) in both treatments.

At flowering stage (50% flowering, 8-week-old plants), physiological measurements were performed in fully expanded leaves of control and stressed plants.

Treatments were maintained until the end of the plant cycle (from June to September) to evaluate grain yield.

Air temperature and relative humidity (RH) were monitored with EasyLog USB Data Loggers (EL-SIE-2+, Lascar Electronics, Erie, PA, USA) during the whole plant growth cycle. The average, minimum and maximum values of temperature and RH are presented in Table 3.

### 4.2. Relative Water Content

Plant water status relative water content (RWC) was determined as described elsewhere [34] following RWC (%) = (FW − DW/TW − DW) × 100] [57]. Briefly, 7 leaf discs of 0.35 cm^2^, from four to five plants per treatment were used. Fresh weight (FW) was assessed immediately after cutting the discs, turgid weight (TW) was determined after overnight water saturation of the discs in a humid chamber at room temperature, and the dry weight (DW) was obtained after 24 h at 80 °C.

### 4.3. Leaf Water Potential

Leaf water potential (y_w_) was determined on the petiole of four to five central leaflets from young fully expanded leaves immediately after excision from each plant using a pressure chamber (Model 1000, PMS Instrument Co., Albany, OR, USA) following [58]. Measurements were taken at pre-dawn and mid-day.

### 4.4. SPAD Measurements

Relative chlorophyll content was obtained with a SPAD (Soil-Plant Analysis Development) meter (SPAD-502 Plus, Konica Minolta, Tokyo, Japan) in the leaf immediately below the leaf used for gas exchange monitoring. Measurements were made before stress induction at the beginning of the flowering stage (T0, BBCH 5) in 5-week-old plants, at end of flowering (BBCH 69) in 7-week-old plants (T1), at BBCH end of fruit development (BBCH 79) in 9-week-old plants (T2), at ripening of about 20% of fruit and seed (BBCH 82) in 10-week-old plants (T3) and at ripening of about 80% of fruit and seed (88) in 11-week-old plants (T4).

### 4.5. Leaf Pigments

Total leaf chlorophylls and carotenoids were extracted from pooled samples of 4 leaf disks (0.35 cm^2^ each) placed in vials containing 10 mL of pure methanol and stored at 4 °C in the dark for 72 h, as described in [59]. Thereafter, the concentration of the extract was determined spectrophotometrically (Shimadzu UV160A, Kyoto, Japan) at 665.2, 652.4 and 470 nm and estimated by using the equations of Lichtenthaler [60].

### 4.6. Electrolyte Leakage Test

For each variety, 12 leaf discs (0.35 cm^2^ each) were cut from expanded leaves, washed with deionized water and floated for 22 h at 25 °C in 10 mL of deionized water. Conductivity values resulting from electrolytes released by cells were read using a conductimeter (Crison GLP 31, Crison Instruments, Barcelona, Spain), at *ca.* 25 °C. Total conductivity was measured after sample exposure to 90 °C in an oven for 2 h, followed by cooling at 25 °C. Membrane injury index (I%) was expressed as a percentage of the total conductivity, according to [59].

### 4.7. Gas Exchange Measurements

Leaf gas exchanges (net photosynthetic rate, P_n_; stomatal conductance, g_s_; transpiration, E) were measured using a portable CO_2_/H_2_O infrared gas analyzer exchange system LI-6400 (LI-Cor, Inc., Lincoln, NE, USA), as described in [61]. An external CO_2_ concentration of *ca.* 370 ppm was used, and chamber block temperature controlled at 25 °C, with artificial light supplied by a “cold” lamp LED type (*ca.* 1000 mmol m^−2^ s^−1^). The parameters were calculated according to the equations of [62]. Instantaneous water use efficiency (iWUE) was estimated as P_n_/E. Measurements were carried out in the morning (10:00–12:00 a.m.). For each parameter, the mean value of three measurements (minimum) is presented.

### 4.8. Chlorophyl Fluorescence Measurements

Chlorophyll (Chl) a fluorescence parameters were obtained, on the same leaves of SPAD measurements, using a FluorPen FP110/D (PSI, Drásov, Czech Republic). The Fv/Fm and Fv’/Fm’ represented the maximal photochemical efficiency of PSII and the actual PSII efficiency of energy conversion under light exposure, respectively. Fv/Fm and Fv’/Fm’ were obtained under dark-adapted (30 min) or photosynthetic steady-state conditions, respectively.

### 4.9. Yield

At the end of the cycle pods were harvested at full maturation stage (complete drying) and threshed manually. The number of pods per plant, number of grains per pod, the weight of 10 grains and total weight of grain per plant were obtained per variety, after oven drying for 35 °C for 72 h.

### 4.10. Soluble Sugars Determination

Soluble sugars (sucrose, fructose, glucose, and sorbitol) were determined in approximately 400 mg of powdered frozen leaf material, based on the method previously described [63] with alterations as in [64]. Briefly, the samples were homogenized in 4 mL of cold H_2_O with 50 mg of polyvinylpolypyrrolidone, left to extract for 20 min on ice at 100 rpm and centrifuged (12,000× *g*, 5 min, 4 °C). The supernatant was boiled to denature the proteins (3 min), placed on ice (6 min) and centrifuged again. The obtained clear solution was then filtered (0.45 µm, nylon) before the injection of a 50 μL aliquot into an HPLC system equipped with a refractive index detector (Model 2414, Waters, Milford, MA, USA). The separation of sugars was performed using a SugarPak 1 column (300 × 6.5 mm, Waters) at 90 °C, with H_2_O as the eluent (containing 50 mg EDTA-Ca L^−1^ H_2_O) and a flow rate of 0.5 mL min^−1^ for 22 min. Standard curves were used for the quantification of each sugar.

### 4.11. Statistical Analysis

ANOVA (*p* < 0.05) was applied using IBM SPSS Statistics 25 program followed by Tukey’s test for mean comparison, and a regression analysis. Different letters express significant differences between landrace (a,b,c) or between control and stress in the same genotype (r,s).

## Figures and Tables

**Table 1:** Relative water content (RWC), injury index (I), leaf water potential at pre-dawn (Ψ_PD_) and mid-day (Ψ_MD_) and degree of isohydricity (σ) of cowpea under well-watered (WW) and water deficit (WD) conditions. Values represent mean ± SE (*n* = 5). Different letters mean significant differences between varieties (a,b) and between treatments for each variety (r,s), (ANOVA, *p* < 0.05).

Variety	RWC (%)		I (%)		Ψ_PD_ (MPa)		Ψ_MD_ (MPa)		σ (Ψ_PD_/Ψ_MD_)
	WW	WD	WW	WD	WW	WD	WW	WD	
CV	88.7 ± 0.70 ^ar^	79.5 ± 2.67 ^as^	11.0 ± 0.88 ^ar^	12.7 ± 0.84 ^abr^	−0.16 ± 0.02 ^ar^	−0.35 ± 0.02 ^as^	−0.64 ± 0.08 ^br^	−0.80 ± 0.06 ^ar^	0.4064
L1	88.2 ± 1.06 ^ar^	81.3 ± 1.66 ^as^	8.7 ± 0.50 ^abs^	10.9 ± 0.53 ^abr^	−0.17 ± 0.01 ^ar^	−0.42 ± 0.02 ^as^	−0.38 ± 0.05 ^ar^	−0.83 ± 0.06 ^as^	1.6743
L2	91.2 ± 0.41 ^ar^	79.5 ± 1.10 ^as^	7.3 ± 0.41 ^bs^	13.3 ± 1.10 ^ar^	−0.16 ± 0.02 ^ar^	−0.39 ± 0.02 ^as^	−0.52 ± 0.05 ^abr^	−0.86 ± 0.08 ^as^	1.2231
L3	89.2 ± 0.92 ^ar^	83.4 ± 1.11 ^as^	6.7 ± 0.28 ^bs^	10.0 ± 0.56 ^br^	−0.14 ± 0.02 ^ar^	−0.43 ± 0.06 ^as^	−0.53 ± 0.03 ^abr^	−0.99 ± 0.10 ^as^	1.3191
L4	90.9 ± 1.27 ^ar^	80.1 ± 0.67 ^as^	9.8 ± 0.43 ^as^	11.5 ± 0.37 ^abr^	−0.17 ± 0.01 ^ar^	−0.42 ± 0.03 ^as^	−0.48 ± 0.06 ^abr^	−0.78 ± 0.08 ^ar^	1.0585

**Table 2:** Effect of water deficit treatment, cowpea genotype and their interaction on relative water content (RWC), injury index (I), leaf water potential at pre-dawn (Ψ_PD_) and mid-day (Ψ_MD_), photosynthesis (Pn), leaf stomatal conductance (gs), internal CO_2_ concentration (Ci), instantaneous water use efficiency (iWUE), maximal photochemical efficiency of PSII of dark-adapted leaves (Fv/Fm), photochemical efficiency of PSII under photosynthetic steady-state conditions (Fv’/Fm’), total chlorophyll, total carotenoids, sum of sugar content of leaves, number of pods per plant (NPPl), number of grains per pod (NGPo), number of grains per plant (NGPl) and weight of 10 grains.

	Treatment	Variety	Variety * Treatment
RWC (%)	***	n.s.	n.s.
I (%)	***	**	n.s.
Ψ_PD_ (MPa)	***	n.s.	n.s.
Ψ_MD_ (MPa)	***	n.s.	n.s.
Pn (µmol m^−2^ s^−1^)	***	n.s.	n.s.
gs (mmol m^−2^ s^−1^)	***	n.s.	n.s.
Ci (µL L^−1^)	***	n.s.	n.s.
iWUE (mmolCO_2_/mol^−1^ H_2_O)	n.s.	n.s.	*
Fv/Fm	***	*	*
Fv’/Fm’	n.s	*	n.s.
Chlorophyll (µg mg^−1^ DW)	***	n.s.	**
Carotenoids (µg mg^−1^ DW)	***	n.s.	**
Sum Sugars (mg g^−1^ DW)	*	n.s.	*
NPPl (number)	***	***	n.s.
NGPo (number)	***	*	n.s.
NGPl (number)	***	***	**
Weight 10 grains (g)	n.s.	***	n.s.

ns—not significant; *—significant (*p* < 0.05); **—very significant (*p* < 0.01); ***—highly significant (*p* < 0.001).

**Table 3:** Temperature and relative humidity in the semi-controlled greenhouse during plant growth.

	Temperature (°C)	Relative Humidity (%)
Average	24.6	63.2
Maximum	46.9	89.4
Minimum	13.9	22.7

## Data Availability

Data is contained within the article.

## References

[1] Graham P.H., Vance C.P. (2003). Legumes: Importance and constrains to greater use. Plant Physiol..

[2] Oritz R. (1998). Cowpeas from Nigeria: A silent food revolution. Outlook Agric..

[3] Tariku S. (2018). Breeding cowpea *Vigna unguiculata* L. Walp for quality traits. Ann. Rev. Res..

[4] Dadson R.B., Hashem F.M., Javaid I., Allen A.L., Devine T.E. (2005). Effect of water stress on yield of cowpea (*Vigna unguiculata* L. Walp.) genotypes in the Delmarva region of the United States. J. Agron. Crop. Sci..

[5] Zhu J.K. (2002). Salt and drought stress signal transduction in plants. Annu. Rev. Plant Biol..

[6] Acuña H., Inostrosza L., Sanchez M.P., Tapia G. (2010). Drought-tolerant naturalized populations of *Lotus tenuis* for constrained environments. Acta Agric. Scand. Sect. B—Soil Plant Sci..

[7] Inès S., Talbi O., Nasreddine Y., Rouached A., Gharred J., Jdey A., Hanana M., Abdelly C. (2021). Drought tolerance traits in Medicago species: A review. Arid Land Research and Management.

[8] Chaves M.M. (1991). Effects of water deficit on carbon assimilation. J. Exp. Bot..

[9] Lawlor D.W., Cornic G. (2002). Photosynthetic carbon assimilation and associated metabolism in relation to water deficits in higher plants. Plant Cell Environ..

[10] Baath G.S., Rocateli A.C., Kakani V.G., Singh H., Northup B.K., Gowda P.H., Katta J.R. (2020). Growth and physiological responses of three warm-season legumes to water stress. Sci. Rep..

[11] Nunes C., Araujo S.S., Silva J.M., Fevereiro M.P.S., Silva A.B. (2008). Physiological responses of the legume model *Medicago truncatula cv. Jemalong* to water deficit. Environ. Exp. Bot..

[12] Carvalho M., Lino-Neto T., Rosa E., Carnide V. (2017). Cowpea: A legume crop for a challenging environment. J. Sci. Food Agric..

[13] Sperry J.S., Hacke U.G., Oren R., Comstock J.P. (2002). Water deficits and hydraulic limits to leaf water supply. Plant Cell Environ..

[14] Jiang J., Su M., Chen Y., Gao N., Jiao C., Sun Z., Li F., Wang C. (2013). Correlation of drought resistance in grass pea (*Lathyrus sativus*) with reactive oxygen species scavenging and osmotic adjustment. Biologia.

[15] Carvalho M., Castro I., Moutinho-Pereira J., Correia C., Egea-Cortines M., Matos M., Rosa E., Carnide V., Lino-Neto T. (2019). Evaluating stress responses in cowpea under drought stress. J. Plant Physiol..

[16] Zegaoui Z., Planchais S., Cabassa C., Djebbar R., Belbachir O.A., Carol P. (2017). Variation in relative water content, proline accumulation and stress gene expression in two cowpea landraces under drought. J. Plant Physiol..

[17] Pinheiro C., Passarinho J.A., Ricardo C.P. (2004). Effect of drought and rewatering on the metabolism of *Lupinus albus* organs. J. Plant Physiol..

[18] Nadeem M., Li J., Yahya M., Sher A., Ma C., Wang X., Qiu L. (2019). Research progress and perspective on drought stress in legumes: A review. Int. J. Mol. Sci..

[19] Lazaridi E., Ntatsi G., Savvas D., Bebeli P.J. (2016). Diversity in cowpea (*Vigna unguiculata* (L.) Walp.) local populations from Greece. Genet. Resour. Crop Evol..

[20] Tosti N., Negri V. (2005). On-going on-farm microevolutionary processes in neighbouring cowpea landraces revealed by molecular markers. Theor. Appl. Genet..

[21] Martínez-Vilalta J., Poyatos R., Aguadé D., Retana J., Mencuccini M. (2014). A new look at water transpot regulation in plants. New Phytol..

[22] Pushpavalli R., Zaman-Allah M., Turner N.C., Baddam R., Rao M.V., Vadez V. (2015). Higher flower and seed number leads to higher yield under water stress conditions imposed during reproduction in chickpea. Funct. Plant Biol..

[23] Ahmed F.E., Suliman A.S.H. (2010). Effect of water stress applied at different stages of growth on seed yield and water-use efficiency of cowpea. Agric. Biol. J. North Amer..

[24] Delmer D.P. (2005). Agriculture in the developing world: Connecting innovations in plant research to downstream applications. Proc. Natl. Acad. Sci. USA.

[25] Parry M.L., Canziani O.F., Palutikof J.P., van der Linden P.J., Hanson C.E., IPCC (2007). Climate Change 2007: Impacts, Adaptation and Vulnerability. Contribution of Working Group II to the Fourth Assessment Report of the Intergovernmental Panel on Climate Change.

[26] Shukla P.R., Skea J., Calvo Buendia E., Masson-Delmotte V., Pörtner H.-O., Roberts D.C., Zhai P., Slade R., Connors S., IPCC (2019). Climate Change and Land: An IPCC Special Report on Climate Change, Desertification, Land Degradation, Sustainable Land Management, Food Security, and Greenhouse Gas Fluxes in Terrestrial Ecosystems.

[27] Farooq M., Farooq M., Hussain M., Siddique K.H.M. (2014). Drought stress in wheat during flowering and grain filling periods. Crit. Rev. Plant Sci..

[28] Frahm M.A., Rosas J.C., Mayek-Pérez N., López-Salinas E., Acosta-Gallegos J.A., Kelly J.D. (2004). Breeding beans for resistance to terminal drought in the lowland tropics. Euphytica.

[29] Beebe S.E., Rao I.M., Cajiao C., Grajales M. (2008). Selection for drought resistance in common bean also improves yield in phosphorus limited and favorable environments. Crop Sci..

[30] Torres A.M., Avila C.M., Gutierrez N., Palomino C., Moreno M.T., Cubero J.I. (2010). Marker-assisted selection in faba bean (*Vicia faba* L.). Field Crop Res..

[31] Amede T., Schubert S. (2003). Mechanisms of drought resistance in grain legumes isosmotic adjustment. Ethiop. J. Sci..

[32] Novick K.A., Konings A.G., Gentine P. (2019). Beyond soil water potential: An expanded view on isohydricity including land–atmosphere interactions and phenology. Plant Cell Environ..

[33] Tardieu F., Simonneau T. (1998). Variability among species of stomatal control under fluctuating soil water status and evaporative demand: Modelling isohydric and anisohydric behaviours. J. Exp. Bot..

[34] Scotti-Campos P., Pham-Thi A.T., Semedo J.N., Pais I.P., Ramalho J.C., Matos M.C. (2013). Physiological responses and membrane integrity in three *Vigna* genotypes with contrasting drought tolerance. Emir. J. Food Agric..

[35] Campos P.S., Ramalho J., Silva M.J., Lauriano J.A., Matos M.C. (1999). Effects of drought on photosynthetic performance and water relations of four *Vigna* genotypes. Photosynthetica.

[36] Blum A. (2015). Towards a conceptual ABA ideotype in plant breeding for water limited environments. Funct. Plant Biol..

[37] Chaves M.M., Zarrouk O., Francisco R., Costa J.M., Santos T., Regalado A.P., Rodrigues M.L., Lopes C.M. (2010). Grapevine under deficit irrigation: Hints from physiological and molecular data. Ann. Bot..

[38] Chaves M.M., Costa J.M., Zarrouka O., Pinheiro C., Lopes C.M., Pereira J.S. (2016). Controlling stomatal aperture in semi-arid regions—The dilemma of saving water or being cool?. Plant Sci. J..

[39] Dow G.J., Berry J.A., Bergmann D.C. (2014). The physiological importance of developmental mechanisms that enforce proper stomatal spacing in *Arabidopsis thaliana*. New Phytol..

[40] Nautiyal P.C., Nageswara Rao R.C., Joshi Y.C. (2002). Moisture deficit-induced changes in leaf-water content, leaf carbon exchange rate and biomass production in groundnut cultivars differing in specific leaf area. Field Crop. Res..

[41] Jones H.G. (1998). Stomatal control of photosynthesis and transpiration. J. Exp. Bot..

[42] Cornic G. (2000). Drought stress inhibits photosynthesis by decreasing stomatal aperture—Not by affecting ATP synthesis. Trends Plant Sci..

[43] Flexas J., Medrano H. (2002). Drought-inhibition of photosynthesis in C3 plants: Stomatal and non-stomatal limitations revisited. Ann. Bot..

[44] Lawlor D.W. (2002). Limitation to photosynthesis in water-stressed leaves: Stomata vs. metabolism and the role of ATP. Ann. Bot..

[45] Sharma A., Kumar V., Shahzad B., Ramakrishnan M., Sidhu G.P., Bali A.S., Handa N., Kapoor D., Yadav P., Khanna K. (2020). Photosynthetic response of plants under different abiotic stresses: A review. J. Plant Growth Regul..

[46] Lopez F.B., Chauhan Y.S., Johansen C. (1997). Effects of timing of drought stress on leaf area development and canopy light interception of short-duration pigeonpea. J. Agron. Crop Sci..

[47] Hayatu M., Mukhtar F.B. (2010). Physiological responses of some drought resistant cowpea genotypes (*Vigna unguiculata* (L.) Walp) to water stress. Bayero J. Pure Appl. Sci..

[48] Fang Y., Xiong L. (2015). General mechanisms of drought response and their application in drought resistance improvement in plants. Cell. Mol. Life Sci..

[49] Muchero W., Roberts P.A., Diop N.N., Drabo I., Cisse N., Close T.J., Muranaka S., Baukar O., Ehlers J.D. (2013). Genetic architecture of delayed senescence, biomass, and grain yield under drought stress in cowpea. PLoS ONE.

[50] Kumudini S. (2002). Trials and tribulations: A review of the role of assimilate supply in soybean genetic yield improvement. Field Crop. Res..

[51] Ismail A.M., Hall A.E., Ehlers J.D. (2000). Delayed leaf senescence and heat tolerance traits mainly are independently expressed in cowpea. Crop Sci..

[52] Ronghua L., Pei-guo G., Baum M., Grando S., Ceccarelli S. (2006). Evaluation of chlorophyll content and fluorescence parameters as indicators of drought tolerance in barley. Agric. Sci. China.

[53] Hamidou F., Zombre G., Braconnier S. (2007). Physiological and biochemical responses of cowpea genotypes to water stress under glasshouse and field conditions. J. Agron. Crop Sci..

[54] Cruz de Carvalho M.H., Laffray D., Louguet P. (1998). Comparison of the physiological responses of *Phaseolus vulgaris* and *Vigna unguiculata* cultivars when submitted to drought conditions. Environ. Exp. Bot..

[55] Iseki K., Takahashi Y., Muto C., Naito K., Tomooka N. (2018). Diversity of drought tolerance in the genus vigna. Front. Plant Sci..

[56] Dumroese R.K., Montville M.E., Pinto J.R. (2015). Using container weights to determine irrigation needs: A simple method. Nativ. Plants J..

[57] Barrs H.D. (1968). Effect of cyclic variations in gas exchange under constant environmental conditions on the ratio of transpiration to net photosynthesis. Physiol. Plant..

[58] Scholander P.F., Hammel H.T., Bradstreet E.D., Hemmingsen E.A. (1965). Sap pressure in vascular plants. Science.

[59] Scotti-Campos P., Semedo J.N., Pais I.P., Oliveira M., Passarinho J., Santos M., Almeida A.S., Costa A.R., Pinheiro N., Bagorro C. (2015). Physiological responses to drought in four developed *Triticum aestivum* groups. Emir. J. Food Agric..

[60] Lichtenthaler H.K. (1987). Chlorophylls and carotenoids: Pigments of photosynthetic biomembranes. Methods Enzymol..

[61] Semedo J.N., Rodrigues A.P., Lidon F.C., Pais I.P., Marques I., Gouveia D., Armengaud J., Silva M.J., Martins S., Semedo M.C. (2021). Intrinsic non-stomatal resilience to drought of the photosynthetic apparatus in *Coffea spp.* is strengthened by elevated air [CO_2_]. Tree Physiol..

[62] Caemmerer S., Farquhar G.D. (1981). Some relationships between the biochemistry of photosynthesis and the gas exchange of leaves. Planta.

[63] Damesin C., Lelarge C. (2003). Carbon isotope composition of current year shoots from *Fagus sylvatica* in relation to growth, respiration and use of reserves. Plant Cell Environ..

[64] Ramalho J.C., Rodrigues A.P., Semedo J.N., Pais I.P., Martins L.D., Simões-Costa M.C., Leitão A.E., Fortunato A.S., Batista-Santos P., Palos I. (2013). Sustained photosynthetic performance of *Coffea spp.* under long-term enhanced [CO_2_]. PLoS ONE.

